# Supplementary material for: The Matricellular Protein Hevin Is Involved in Alcohol Use Disorder
Source: Biomolecules. 2023 Jan 25;13(2):234. doi: 10.3390/biom13020234 (PMC9953008; doi:10.3390/biom13020234)
Supplement: Supplementary file 1 [file biomolecules-13-00234-s001.zip › biomolecules-2156296-supplementary.pdf]

Supplementary Table S1. Demographic characteristics, postmortem delay (PMD), storage time of the sample, cause of death, psychiatric diagnosis and toxicological findings of the subjects included in the study. AUD, alcohol use disorder; C, control; D, depression; M, male; F, female; CRF, cardiorespiratory failure.

Case	Sex	Age (Years)	PMD (Hours)	Storage Time (Months)	Cause of Dead	Psychiatric Diagnosis	Drugs in Blood
AUD1	M	59	22	11	Natural/ Cirrhosis	AUD	(-)
C1	M	57	3	18	Accident/ Crushing	Control	(-)
D1	M	55	20	95	Suicide/ Train	Depression	Antidepressants
AUD2	M	59	19	159	Natural/ CRF	AUD	(-)
C2	M	58	16	201	Accident/ Traffic	Control	(-)
D2	M	60	18	170	Suicide/ Hanging	Depression	Antidepressants
AUD3	M	61	34	20	Natural/ CRF	AUD	Ethanol
C3	M	62	9	45	Natural/ CRF	Control	(-)
D3	M	62	15	96	Suicide/ Knife	Depression	Antidepressants
AUD4	M	51	23	23	Natural/ CRF	AUD	(-)
C4	M	51	14	108	Accident/ Traffic	Control	Ethanol
D4	M	51	24	200	Suicide/ Hanging	Depression	Antidepressants
AUD5	M	55	11	36	Natural/ CRF	AUD	(-)
C5	M	56	13	96	Natural/ CRF	Control	(-)
D5	M	56	3	128	Natural/ Thromboembolism	Depression	Antidepressants
AUD6	M	61	27	162	Natural/ CRF	AUD	(-)
C6	M	61	23	202	Accident/ Traffic	Control	(-)
D6	M	60	24	111	Suicide/ Jumping	Depression	Antidepressants
AUD7	M	52	17	85	Natural/ CRF	AUD	(-)
C7	M	54	16	112	Accident/ Work	Control	(-)
D7	M	50	23	143	Suicide/ Gun	Depression	Antidepressants
AUD8	M	47	28	88	Natural/ Hemorrhage	AUD	Ethanol
C8	M	46	24	129	Natural/CRF	Control	(-)
D8	M	49	27	18	Natural/CRF	Depression	Antidepressants
AUD9	F	50	14	95	Natural/ Hemorrhage	AUD	Ethanol
C9	F	50	10	129	Natural/CRF	Control	(-)
D9	F	49	19	164	Suicide/ Jumping	Depression	Antidepressants
AUD10	F	38	22	165	Natural/ CRF	AUD	(-)
C10	F	36	20	123	Accident/ Train	Control	(-)
D10	F	36	32	152	Suicide/ Drug intoxication	Depression	Anxiolytics/ Antidepressants
AUD11	F	71	16	125	Natural/ CRF	AUD	(-)
C11	F	71	22	155	Accident/ Traffic	Control	(-)
D11	F	70	7	92	Suicide/ Jumping	Depression	Antidepressants
AUD12	M	64	9	104	Natural/ CRF	AUD	Ethanol
C12	M	64	22	46	Natural/ CRF	Control	(-)
D12	M	65	12	145	Suicide/ Hanging	Depression	Antidepressants
AUD13	M	73	2	168	Natural/ CRF	AUD	Ethanol
C13	M	73	10	202	Accident/ Jumping	Control	(-)
D13	M	73	11	39	Suicide/ Hanging	Depression	Antidepressants
AUD14	M	52	27	114	Natural/ CRF	AUD	(-)
C14	M	51	19	101	Accident/ Traffic	Control	(-)
D14	M	53	22	87	Suicide/ Hanging	Depression	Antidepressants
AUD15	M	49	10	115	Natural/ CRF	AUD	Ethanol
C15	M	48	10	123	Natural/CRF	Control	(-)
D15	M	50	24	118	Suicide/ Gun	Depression	Antidepressants
AUD16	M	56	24	129	Natural/ CRF	AUD	Ethanol
C16	M	55	22	130	Natural/CRF	Control	(-)

D16	M	56	22	119	Suicide/ Gun	Depression	Antidepressants
AUD17	F	54	7	142	Natural/ CRF	AUD	Ethanol
C17	F	51	10	84	Natural/CRF	Control	(-)
D17	F	54	18	169	Suicide/ Hanging	Depression	Antidepressants
AUD18	M	60	6	143	Natural/ CRF	AUD	(-)
C18	M	60	19	87	Natural/ CRF	Control	(-)
D18	M	61	5	198	Natural/ Aneurism	Depression	Antidepressants
AUD19	M	46	11	149	Natural/ Hemorrhage	AUD	(-)
C19	M	47	17	205	Natural/ CRF	Control	(-)
D19	M	47	18	167	Suicide/ Hanging	Depression	Antidepressants
AUD20	F	44	11	163	Natural/ CRF	AUD	(-)
C20	F	45	12	130	Natural/ CRF	control	(-)
D20	F	43	24	143	Natural/CRF	Depression	Antidepressants
AUD21	M	53	25	158	Natural/ CRF	AUD	(-)
C21	M	54	23	159	Accident/ Jumping	control	(-)
D21	M	53	39	191	Suicide/ Gun	Depression	Antidepressants
AUD22	M	65	5	159	Natural/ CRF	AUD	Ethanol
C22	M	67	22	166	Natural/ CRF	control	(-)
D22	M	65	11	94	Suicide/ Drug intoxication	Depression	Anxiolytics/ Antidepressants
AUD23	F	50	23	159	Natural/ CRF	AUD	Ethanol
C23	F	45	8	21	Natural/ Hemorrhage	Control	(-)
D23	F	49	18	142	Suicide/ Submersion	Depression	Antidepressants
AUD24	M	57	17	147	Natural/ CRF	AUD	(-)
C24	M	55	20	37	Accident/ Mountain	Control	(-)
D24	M	57	21	92	Suicide/ Jumping	Depression	Antidepressants
AUD25	F	46	19	156	Natural/ CRF	AUD	Ethanol
C25	F	43	3	40	Accident/ Traffic	Control	(-)
D25	F	45	21	89	Suicide/ Hanging	Depression	Antidepressants
AUD	7F/18M	55±2	17±2	119±10			
C	7F/18M	54±2	15±1	114±12			
D	7F/18M	55±2	19±2	127±9			

Supplementary Table S2. Details of the primary and secondary antibodies used in western blot assays. PFC, prefrontal cortex; HIP, hippocampus; CAU, caudate nucleus; CB, cerebellum; FC, frontal cortex; NAc, nucleus accumbens; CPU, dorsal striatum; AMY, amygdala.

Sample	Primary Ab	Primary Ab Dilution	Secondary Ab	Secondary Ab Dilution
**Human brain (PFC, HIP, CAU, CB)**	Goat anti-human hevin (R&D systems, AF2728)	1:3000	Alexa Fluor® 680 conjugated donkey anti-goat (Life Technologies, A21084)	1:5000
	Mouse anti- β-actin (Sigma-Aldrich, A1978)	1:100.000	DyLight™ 800 conjugated donkey anti-mouse (Rockland, 610-745-002)	1:5000
**Mouse brain (FC, NAc, CPu, HIP, AMY) and plasma**	Goat anti-mouse hevin (R&D systems, AF2836)	1:2000	Alexa Fluor® 680 conjugated donkey anti-goat (Life Technologies, A21084)	1:5000
	Rabbit anti- β-actin (Abcam, Ab8227)	1:5000	DyLight™ 800 conjugated donkey anti-rabbit (Rockland, 611-745-127)	1:5000
